# Molecular Mechanisms of Luteolin Induced Growth Inhibition and Apoptosis of Human Osteosarcoma Cells 

**Published:** 2015

**Authors:** Yonghong Wang, Daliang Kong, Xinwei Wang, Xiaoxiong Dong, Yingying Tao, Haiyang Gong

**Affiliations:** a *Department of Orthopedics, 86 Hospital of PLA, Dangtu, Anhui 243100, China.*; b *Department of Orthopedics,Japan Union Hospital of Jilin University, Changchun130033,China. *; c *Department of Pharmacy, Jinling Hospital, Nanjing, 210002, China.*; d *Department of Orthopedics, Jingdu Hospital, Nanjing 210002, China.*

**Keywords:** Luteolin, Osteosarcoma cells, Proliferation, Apoptosis, Anti-oxidant

## Abstract

Luteolin is a flavone in medicinal plants as well as some vegetables and spices. It is a natural anti-oxidant with less pro-oxidant potential but apparently with a better safety profile. The purpose of this study was to investigate the molecular mechanism of luteolin-mediated apoptosis of MG-63 human osteosarcoma cells. MTT assay kit was employed to evaluate the effects of luteolin on MG-63 cells proliferation. Then, we performed Annexin V-FITC/PI to analyze the apoptotic rate of the cells. Furthermore, the inhibitory effects of luteolin on the expressions of BCL-2, BAX, Caspase-3 and Survivin were detected by Western blotting. As expected, luteolin (0.5, 2.5, 12.5 µg/mL) inhibited the growth of MG-63 cells by inhibiting cell proliferation and inducing cell apoptosis. Western blotting demonstrated that luteolin (0.5, 2.5, 12.5 µg/mL) inhibited the expressions of BCL-2, Caspase-3 and Survivin, and promoted the expression of BAX in MG-63 cells with a concentration dependent way. Luteolin can inhibit osteosarcoma cell proliferation and induce apoptosis effectively in a dose dependent manner through down-regulating the expression of BCL-2, Caspase-3 and Survivin proteins levels and up-regulating the expression of BAX protein level. These findings indicated that luteolin may be used as a novel herbal medicine for the treatment of osteosarcoma.

## Introduction

Osteosarcoma is a primary bone malignancy with high rates of metastasis, mortality and disability, and this disease usually occurs in children and adolescents (1). The most common treatments for osteosarcoma are surgery, chemotherapy and biotherapy. However, the prognosis of osteosarcoma is still need to be further investigated because of the high degree of malignancy, rapid disease progression and early metastasis (2). Though the traditional treatments have been improved (such as surgery and chemotherapy), the efficacies of new therapy methods are still needed to be evaluated to improve survival rate. With the developments of natural medicinal chemistry and molecular biology, the fundamental research of osteosarcoma has also been promoted. Traditional Chinese herbal extract is one of the research hotspots nowadays (3). The therapeutic strategies that combine traditional Chinese medicine or its active ingredients with surgery and chemotherapy treatment can reduce the toxicities of traditional therapy and drugs, leading to the improvements of patients' immunity and compliance (4-6).

BCL-2 (B-cell lymphoma 2), the founding member of the BCL-2 family, participates in the regulation of cell apoptosis (7). BAX (Bcl-2-associated X protein), a member of the BCL-2 protein family, promotes cell apoptosis by binding to and antagonizing the BCL-2 protein. The BAX gene is the first identified pro-apoptotic Bcl-2 family member (8). Damage to the BCL-2 gene has been identified as a cause of many cancers, including melanoma, breast cancer and prostate cancer. The Caspase 3 protein, a member of the cysteine-aspartic acid protease (caspase) family, is a crucial mediator of programmed cell death (9). Survivin, a member of the inhibitor of apoptosis (IAP) family, could inhibit caspase activation to negative regulate apoptosis or programmed cell death. Luteolin (3',4',5,7-tetrahydroxyflavone) is a natural flavonoid compound extracted from a variety of traditional Chinese medicines, such as *Lonicerajaponica*, *Schizonepetatenuifolia* and *Ajugadecumbens Thunb *(2). The important structure features of luteolin, including 2-3 carbon double bond and hydroxyl moieties at carbons 5, 7, 3’, and 4’ positions, are associated with the biological and biochemical activities of luteolin (10, 11). The anticancer property of luteolin is related to the biological effects of luteolin, such as angiogenesis, anti-inflammatory activity, inhibition of cell proliferation, induction of apoptosis and metastasis (12). Previous reports have indicated that luteolin has various biological activities, such as immunoregulation (13), anti-oxidant (14), anti-inflammation (15), cardiovascular protection (16) and enzyme inhibition (17). In recent years, luteolin has been proved to inhibit the proliferation of leukemia cells (18) and invasion of prostate cancer cells (19, 20). Luteolin also could induce the apoptosis of hepatoma cells (21, 22) and lung cancer cells    (23). What’s more, luteolin has anti-proliferative, chemosensitizing and radiosensitization effects on gastric cancer cells (24, 25). However, the effects of luteolin on human osteosarcoma cells are still unknown.

This study was undertaken to investigate the effects and molecular mechanisms of luteolin on the proliferation and apoptosis of osteosarcoma cells. We established osteosarcoma cell model *in-vitro* and intervened osteosarcoma MG-63 cells with luteolin. Not only the proliferation and apoptosis of MG-63 cells, but also protein expressions of BCL-2, BAX, Caspase-3 and Survivin were measured. 

## Experimental

*Cell line and culture conditions*

Human osteosarcoma cell line MG-63 was purchased from the Shanghai Institute of Cell Bank, the Chinese Academy of Sciences (Shanghai, China) and grown in Dulbecco's modified eagle medium (DMEM) with 10% (v/v) fetal calf serum, streptomycin (100 U/mL) and penicillin (100 U/mL). Luteolin was purchased from the National Institute for the Control of Pharmaceutical and Biological Products (Beijing, China). DMEM, fetal bovine serum (FBS) and Dimethyl sulfoxide (DMSO) were purchased from Gibco Biotechnology (GIBCO BRL, USA). Cultures were maintained at 37 °C incubator with humidified atmosphere of 5% CO_2_.

*MTT assay*

MTT assay was used to assess the viability of the cells. Methylthiazoletetrazolium (MTT) was purchased from Axygen, Inc (Axygen, USA). Cells (5 × 10^3^/mL) were plated on a 96-well plate. Control group (without drug), zero group (without cells) and luteolin group (0.5, 2.5, and 12.5 µg/mL) were set up in this experiment. The cell counting kit 8 (CCK-8) was purchased from Beyotime institute of Biotechnology (Haimen, Jiangsu, China). After culturing for 48 hours, 10 μL of CCK-8 solution was added to each well. After 2 hours incubation at 37 °C, the optical density (OD) value at 450 nm was measured. The cell survival rate (%) = 1 - [(control well - zero well) - (experimental well - zero well)] / (control well - zero well) × 100%. 

*Annexin V-FITC /PI flow cytometry analysis*

MG-63 Cells (5 × 10^5^/mL) were plated on a 96-well plate. AnnexinV-FITC/PI kit was purchased from Biovision Corp (CA, USA). Control group (without drug) and luteolin group (0.5, 2.5, 12.5 μg/mL) were set up in this experiment. MG-63 cells were harvested and resuspended in binding buffer after being cultured for 24 hours. The percentages of apoptotic or necrotic cells following the effects of hypoxia preconditioning were determined using the Annexin V-FITC/PI Detection Kit according to the manufacturer’s instructions. Briefly, MG-63 cells were washed with cold PBS (phosphate buffer solution) twice, and then resuspended in 100 μL binding buffer with the addition of 5 μL Annexin V-fluorescein isothiocyanate (FITC) and 5 μL propidium iodide (PI). The mixture was kept in dark for 15 min at room temperature, and then 400 μL of binding buffer was added for immediately analysis by flow cytometry.

*Western blotting analysis*

The expression of cellular proteins was evaluated by Western blotting. After treatment for 24 hours, the cells were washed with ice cold PBS, and lysed with radioimmunoprecipitation analysis (RIPA) buffer. After centrifugation, the supernatants were collected and protein concentration was determined by bicinchoninic acid (BCA) protein assay. The enhanced chemiluminescence (ECL) kit BCA protein assay Kit and RIPA lysis buffer were purchased from Beyotime institute of Biotechnology (Haimen, Jiangsu, China). Anti-BCL-2, anti-BAX and anti-Caspase-3 rabbit polyclonal antibodies, and peroxidase-conjugated goat anti-rabbit polyclonal antibody were purchased from Santa Cruz Biotechnology (Santa Cruz, CA, USA). Equal amounts of protein from each sample were separated by sodium dodecyl sulfate polyacrylamide gel electrophoresis (SDS-PAGE). After electrophoresis, proteins were electro-blotted to polyvinylidene fluoride (PVDF) membranes. After blocking with PBST (PBS containing 0.05% Tween 20) containing 5% nonfat milk for 1 hour at room temperature, the membranes were incubated with the primary antibodies (1:1000) of anti-BCL-2, anti-BAX and anti-caspase-3 separately overnight at 4 °C. After being washed three times with TBST (20 mM Tris-Cl, pH 7.5, 150 mM NaCl, 1 g/L Tween20), membranes were incubated with the appropriate secondary peroxidase-conjugated antibodies (1:2000). The membranes were washed three times with TBST after incubation, and visualization was made using an ECL kit. 

*Statistical analysis*

The data were expressed as mean ± SD (standard deviation). Statistical correlation of data was checked for significance by ANOVA and Student's t-test. Differences with P < 0.05 were considered significant. These analyses were performed using SPSS 11.0 software (SPSS Inc., Chicago, IL, USA).

## Results

*Luteolin suppressed the proliferation of human osteosarcoma MG-63 cells*

**Figure 1:**
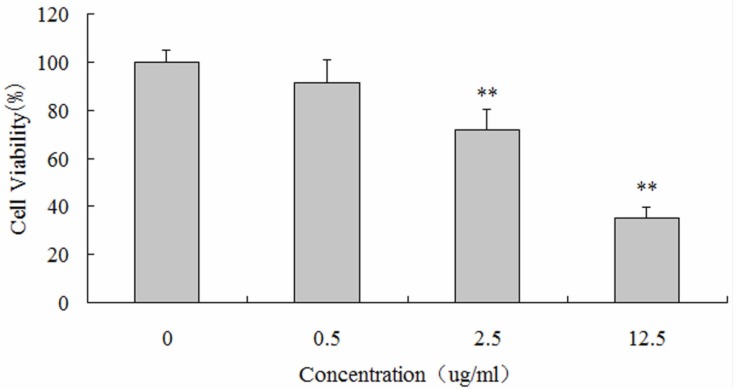
The proliferative inhibition effects of luteolin on human osteosarcoma MG-63 cells. Data are from six independent experiments (∗∗ P < 0.05).

CCK-8 assays were performed to detect the impact of luteolin on the proliferation of MG-63 cells. MG-63 cells were respectively incubated with 0, 0.5, 2.5 and 12.5 µg/mL of luteolin for 48 hours. MG-63 cells incubated with 2.5 and 12.5 µg/mL of luteolin exhibited significantly decreased cellular viability (72.03 ± 8.42%, 35.06 ± 4.56%; P < 0.05), compared with the control group. These results indicated that luteolin treatment evoked growth inhibition of MG-63 cells in a dose-dependent manner (Figure 1). 

*Luteolin induced apoptosis in human osteosarcoma cells*

**Figure 2:**
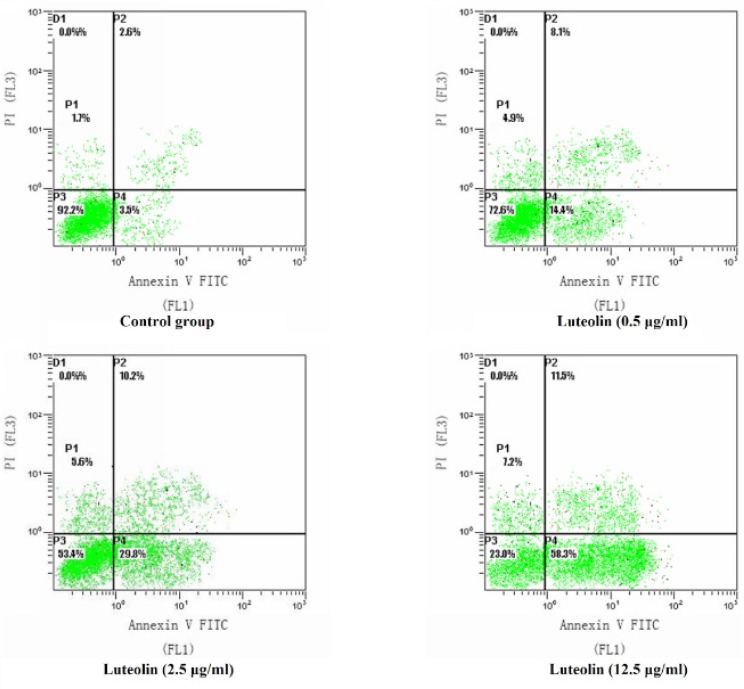
The effect of luteolin on the apoptosis of MG-63 cells by flow cytometry in control and luteolin (0.5 μg/mL, 2.5 μg/mL and 12.5 μg/mL) groups

MG-63 cells were incubated with 0, 0.5, 2.5, 12.5 µg/mL of luteolin for 24 hours and were analyzed by flow cytometry. As shown in Figure 2, the proportions of early apoptotic cells were 3.5%, 14.4%, 29.8% and 58.3%, respectively (Figure 2). These results indicated that luteolin treatment evoked apoptosis of MG-63 cells in a concentration-dependent manner (P < 0.05). 

*The effects of luteolin on the* *protein expressions of BCL-2, BAX and* *Caspase-3*

**Figure 3:**
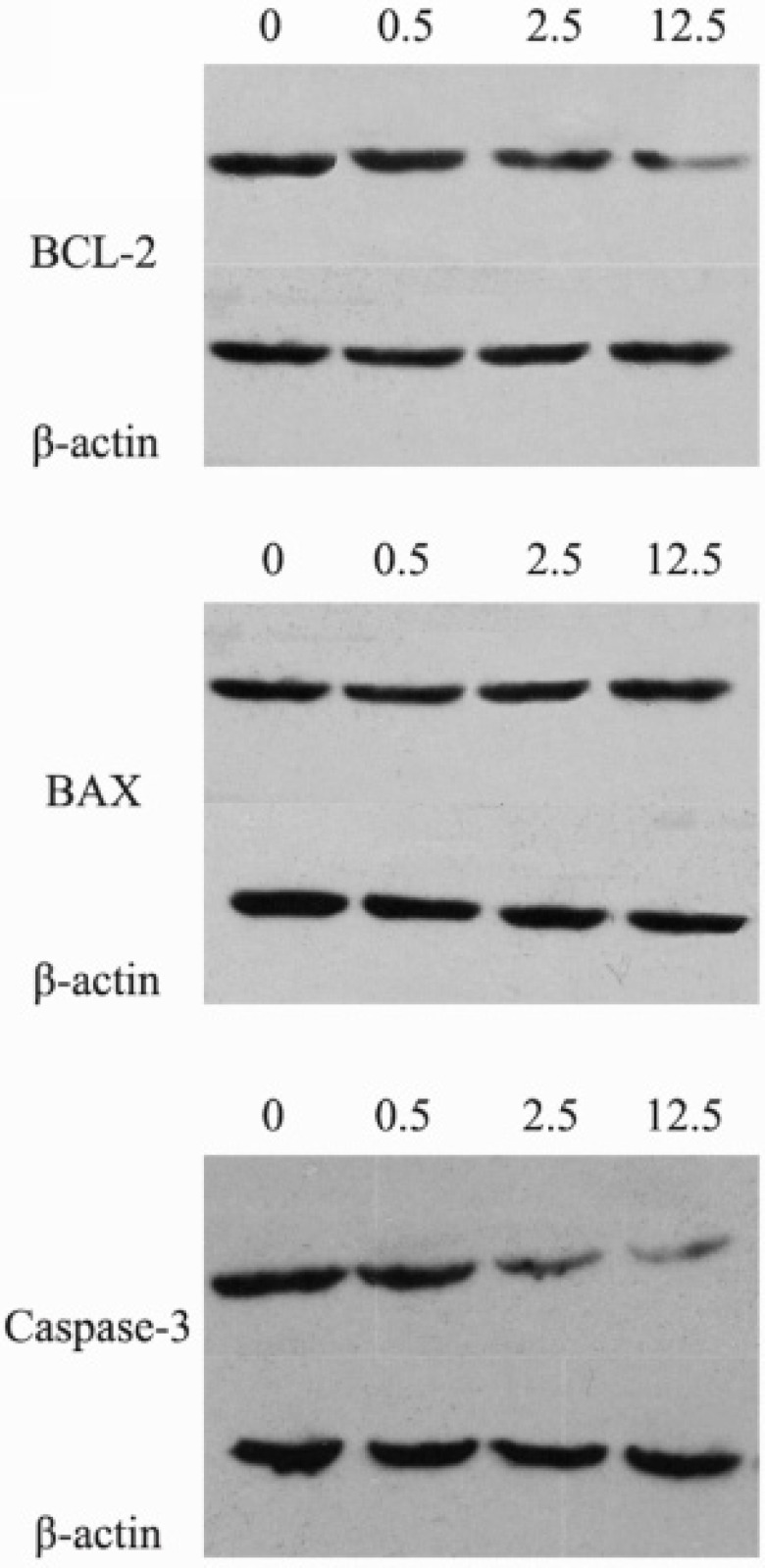
The protein expression levels of BCL-2, BAX and caspase-3 after the treatment of luteolin (0, 0.5, 2.5, 12.5 μg/mL, respectively) for 24 hours. 0: control group; 0.5: Luteolin (0.5 μg/mL); 2.5: Luteolin (2.5 μg/mL); 12.5: Luteolin (12.5 μg/mL)

The protein expression levels of BCL-2, BAX and Caspase-3 were assessed by Western blotting. Compared with the control group, the protein expression levels of BCL-2 and Caspase-3 were significantly decreased, while the expression levels of BAX significantly increased in the luteolin group (Figure 3, P < 0.05). This result indicated that the BAX/BCL-2 ratio was also significantly increased. Therefore, luteolin might induce apoptosis of MG-63 cells through the mitochondrial pathway.

*Luteolin reduced the expression of* *Survivin* *in MG-63 cells*

**Figure 4:**
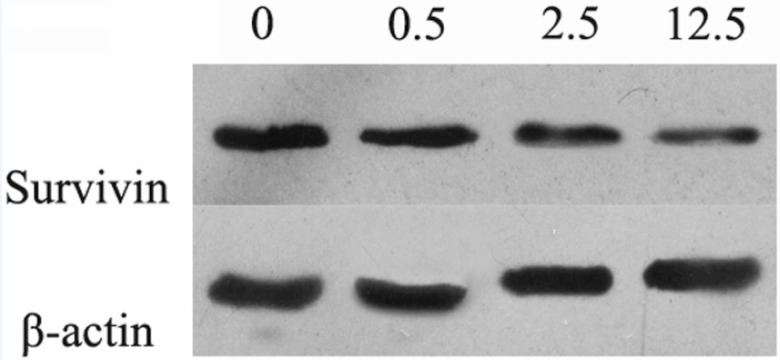
The protein expression level of Survivin after the treatment of luteolin (0, 0.5, 2.5, 12.5 μg/mL, respectively) for 24 hours. 0: control group; 0.5: Luteolin (0.5 μg/mL); 2.5: Luteolin (2.5 μg/mL); 12.5: Luteolin (12.5 μg/mL)

With the increasing concentration of luteolin, the protein expression level of Survivin was down-regulated in a dose dependent manner (Figure 4). Therefore, the apoptosis of MG-63 cells induced by luteolin might be involved with Survivin.

## Discussion

In recent years, studies have shown that many of the herbal medicines have antitumor effects by inhibiting tumor cell proliferation and inducing apoptosis of tumor cells (-). Luteolin, an active flavonoid compound which widely exists in natural medicinal plants, vegetables and fruits (29). Previous studies show that luteolin not only participates in inhibiting tumor cell proliferation, inducing tumor cell apoptosis, influencing tumor cell cycle distribution and inhibiting the formation of new blood vessels in tumor but also can be considered as a tumor cell apoptosis sensitization or antioxidant agents (30). In this study, experimental data demonstrated that the luteolin can strikingly inhibit the proliferation of MG-63 cells with a dosage dependent manner. After 48 hours co-cultured with 12.5 µg/mL luteolin, suppression ratio of MG-63 cells reached up to 64.96 ± 4.56%, which showed that luteolin could inhibit the proliferation of MG-63 cells efficiently. 

Apoptosis, also known as programmed cell death, resulted by the stimulation of many signals inside or outside of cell, plays an important role in the process of cell growth and issue development (31, 32). So far, it has been found that there are three pathways for cell apoptosis, including endoplasmic reticulum pathway (Anoikis apoptosis), mitochondria pathway (intrinsic pathway), and death ligand pathway (extrinsic pathway) (33). The anti-apoptosis protein BCL-2 and pro-apoptosis protein BAX are the two significant regulators for mitochondria pathway (34). The activation of BAX leads to the permeabilization of mitochondrial outer membrane and release of cytochrome C, so as to stimulate caspase protease family and cascade reaction, then result in cell apoptosis (35-37). Therefore, BCL-2, BAX and caspase family protein play important roles in signal pathway for cell apoptosis. BAX can inhibit BCL-2 by forming heterodimer with it, and the ratio relationship of BCL-2 and BAX are the key factors to determinate the inhibition for cell apoptosis (38). Caspase-3 is a kind of aspartic acid specific cysteine protease, which activates as Cleaved Caspase-3 by specific shearing off the inactive subunit in the location of aspartic acid, then take part in the signal transduction of cell apoptosis (39). Through the Annexin V-FITC/PI double staining and Western blotting detection for osteosarcoma MG-63 cell apoptosis and related apoptosis protein expression, we found that the luteolin induced cell apoptosis mechanisms may involve with down-regulation of BCL-2 protein, up-regulation of BAX protein expression, increased BAX/BCL-2 ratio, and the activation of Caspase 3 protein cascade reaction. Luteolin also been reported to induce apoptosis in other types of cancer cells (such as, hepatocellular carcinoma cells, colorectal cancer cells and pancreatic carcinoma cells) through activating Casepase-3, increasing Bax and decreasing BCL-2 protein expression levels (40-42). In this study, we found that Survivin expression was down-regulated and presented dose dependent manner with the concentration of luteolin in MG-63 cells. This suggests that Survivin is a target protein of luteolin which induces osteosarcoma cell apoptosis. Similarly, the growth inhibition and apoptosis of human hepatoma cells and esophageal squamous carcinoma cells induced by luteolin have been found to be related to decreased expression level of Survivin (43, 44). 

Recently studies have shown that some anticancer-drugs could affect cytotoxicity and apoptosis related BAX, BCL-2, and Caspase-3 protein expression (45). Our experiments confirmed that luteolin could promote the apoptosis of MG-63 cells through Survivin dependent pathway and activated Caspase-3 protein's cascade process is one of the possible mechanisms. Until now, the main treatments of osteosarcoma are still with surgery and chemotherapy; traditional chemotherapy drugs have the defects of side effects and drug resistance. Therefore, whether luteolin can overcome these insufficient and has synergies with traditional chemotherapy drugs, and whether its pro-apoptotic effect on tumor cell related to other mechanisms remain to be further studied.

## Conclusions

Our studies demonstrated that luteolin inhibited MG-63 osteosarcoma cell proliferation and induced apoptosis effectively in a dose dependent manner through down-regulating the expression of BCL-2, Caspase-3 and Survivin proteins levels and up-regulating the expression of BAX protein. This might be the important molecular mechanisms of luteolin induced growth inhibition and apoptosis of the MG-63 osteosarcoma cells. Our findings suggest that luteolin may have a therapeutic application in the treatment of human osteosarcoma. 
